# Recommendations for improving the working conditions and cultures of distressed junior doctors, based on a qualitative study and stakeholder perspectives

**DOI:** 10.1186/s12913-022-08728-2

**Published:** 2022-11-10

**Authors:** Johanna Spiers, Farina Kokab, Marta Buszewicz, Carolyn A. Chew-Graham, Alice Dunning, Anna K. Taylor, Anya Gopfert, Maria van Hove, Kevin Rui-Han Teoh, Louis Appleby, James Martin, Ruth Riley

**Affiliations:** 1 School of Health Sciences, Faculty of Health and Medical Sciences, University of Surrey, Guildford, UK; 2 Department of Social Work and Social Policy, School of Social Policy, University of Birmingham, Birmingham, UK; 3 Research Department of Primary Care and Population Health, University College London Medical School, London, UK; 4 School of Medicine, Keele University, Keele, UK; 5 Yorkshire Quality and Safety Research Group, Bradford Institute for Health Research, Bradford Teaching Hospitals NHS Foundation Trust, Bradford, UK; 6 Leeds Institute of Health Sciences, Faculty of Medicine and Health, University of Leeds, Leeds, UK; 7 University of Exeter, Exeter, EX4 4PY UK; 8 Department of Organizational Psychology, Birkbeck, University of London, London, UK; 9 Division of Psychology and Mental Health, School of Medicine, University of Manchester, Manchester, UK; 10 Institute of Applied Health Research, University of Birmingham, Birmingham, UK

**Keywords:** Junior doctors, Mental health, Recommendations, Qualitative research

## Abstract

**Background:**

Doctors, including junior doctors, are vulnerable to greater levels of distress and mental health difficulties than the public. This is exacerbated by their working conditions and cultures. While this vulnerability has been known for many years, little action has been taken to protect and support junior doctors working in the NHS. As such, we present a series of recommendations from the perspective of junior doctors and other relevant stakeholders, designed to improve junior doctors’ working conditions and, thus, their mental health.

**Methods:**

We interviewed 36 junior doctors, asking them for recommendations for improving their working conditions and culture. Additionally, we held an online stakeholder meeting with a variety of healthcare professionals (including junior doctors), undergraduate medical school leads, postgraduate speciality school leads and NHS policymakers where we asked what could be done to improve junior doctors’ working conditions. We combined interview data with notes from the stakeholder discussions to produce this set of recommendations.

**Results:**

Junior doctor participants and stakeholders made organisational and interpersonal recommendations. Organisational recommendations include the need for more environmental, staff and educational resources as well as changes to rotas. Interpersonal recommendations include changes to communication and recommendations for better support and teamwork.

**Conclusion:**

We suggest that NHS policymakers, employers and managers consider and hopefully implement the recommendations set out by the study participants and stakeholders as reported in this paper and that the gold standards of practice which are reported here (such as examples of positive learning environments and supportive supervision) are showcased so that others can learn from them.

**Supplementary Information:**

The online version contains supplementary material available at 10.1186/s12913-022-08728-2.

## Background

Doctors are vulnerable to burnout [1–6], anxiety and depression [7–9], and, in several countries, increased suicide rates [10–14]. Junior doctors face additional stressors as they move from being students to qualified doctors, including the transition from study to work [13], role uncertainty [5, 6, 13, 15], low pay relative to their years of training [15], poor leadership or lack of support [5, 16, 17], contract concerns [5], assessment and training requirements [6], a potentially toxic working environment [17], frequent rotations and – in recent times – COVID-19-related redeployment [18]. It is, therefore, perhaps unsurprising that junior doctors are vulnerable to mental ill-health and burnout [19].

These vulnerabilities have been known for many years [7–9, 16], and yet they persist [20]. While there have been repeated calls for change and interventions to better support doctor wellbeing [21], most interventions for doctors in the UK have focused on the individual [19]. These include mindfulness and resilience training, psychoeducation, promoting healthy lifestyle changes and access to talking therapies [22, 23]. Although there is some evidence for their effectiveness [22, 23], these interventions are limited as they do not address many of the underlying systemic factors contributing to junior doctors’ wellbeing – namely poor working conditions and cultures. Moreover, individual interventions often imply that responsibility for one’s wellbeing lies within the individual and not the system [24].

Best practice guidance on wellbeing interventions emphasises the importance of a participatory approach when taking a more systematic approach [25]. This draws on the experience and expertise of affected workers and corresponding stakeholders to first identify underlying issues and then generate possible solutions to address them. In previous papers from our study, we have presented findings on the experiences of junior doctors working during the COVID-19 pandemic [26], the sources of distress for junior doctors [17] and any protective factors that doctors themselves were employing whilst working in challenging conditions [27]. In this article, we outline specific, tangible recommendations for how junior doctors’ working conditions and cultures could be improved. Given the need to ask those on the frontline about their experiences and ideas, qualitative methods were deemed the most appropriate for this study.

## Method

### Design and data collection

This qualitative study is part of a larger mixed-methods study exploring junior doctors’ perceptions of stress and distress [17, 26, 27]. The study setting was the NHS in England. The data that inform this paper are drawn from traditional qualitative interview data as well as comments and discussions from a stakeholder meeting held on June 25^th^ 2021.

A total of 36 junior doctor research participants took part in semi-structured interviews which explored their experiences; see our previous paper [26] for more detail. Our semi-structured topic guide was modified iteratively as data collection progressed. This guide aimed to capture participants’ experiences, feelings and beliefs about working conditions and cultures which were perceived as stressful or distressing, as well as ideas about how participants’ working conditions could be improved. The guide was informed by the existing literature, input from junior doctors on the study team and patient and public involvement (PPI) consultation exercises conducted prior to obtaining funding. Following conventions for semi-structured interviews [28], questions from the topic guide were followed up with individualised questions exploring topics of interest and importance that came up for each participant. Questions were open and exploratory (for example, *has work impacted your mental health and wellbeing?*). We did not pilot the topic guide as the interviewers were experienced qualitative researchers.

Interviews were conducted either face-to-face, on the telephone or via video call; participants chose the medium and location for their interview. They were given an information sheet about the study and the opportunity to ask any questions before the interview. The interviews took place between November 2019 and February 2021. Since some interviews took place during COVID-19, those conversations included data on the impact of the pandemic, which we have analysed in another paper [26]. A risk protocol was used to ensure appropriate support was provided to participants in the event of the disclosure of suicidal ideation. The in-depth interviews were conducted by JS, a female PhD psychologist with qualitative methods expertise; FK, a female PhD non-clinical researcher from an applied health research background; and RR, a female medical sociologist with qualitative methods expertise. None of the interviewers had a relationship with any of the participants prior to the interviews; however, all worked to establish rapport and warmth during data collection. Relevant field notes were made after each interview.

The recorded interviews were transcribed verbatim and checked for accuracy before analysis. All transcripts were anonymised before discussion within the wider research team. Interviews were between 29 and 103 minutes long (mean = 64 minutes).

Additionally, we presented a summary of previous findings from this project [17, 27] to the online stakeholder meeting in June 2021, where we posed the following questions:

- What power do you, or your organisation, have to make changes?
- How can you, or your organisation, make realistic improvements to prevent work-related distress and support junior doctors?
- What actions can you, or your organisation, take to address the causes of work-related distress identified by this research?

The aim of the stakeholder meeting was to disseminate our research and open a discussion with key NHS workers about how working conditions could be improved for junior doctors. Details of stakeholders’ job roles can be seen below. The meeting was held via Zoom and included a presentation of research findings so far; a Q&A with guest commentators and participants; comments from other stakeholders; small group discussions in breakout rooms; feedback from the small group discussions to the wider group; guest commentators advising on next steps; and closing remarks. Involving stakeholders in the creation and application of research can lead to policy or practice change [29, 30], which is in line with our desire for these recommendations about junior doctors’ working conditions and culture to be examined and implemented by policymakers. Additionally, by working collaboratively alongside stakeholders, we support the creation of a culture of equality, reciprocity and mutuality, inviting policymakers to take an active role in our research [31].

Stakeholders were divided into smaller groups of varying seniority and background disciplines to discuss the above questions. Their responses were fed back to the plenary and discussed further. Detailed notes of the meeting and all discussions were made and circulated to both the stakeholders and the research group. The main meeting itself was also recorded and further notes were made following a viewing of that recording.

### Participants and ethics

Two sets of semi-structured interviews and an online survey were used to explore junior doctors’ experiences of working culture and conditions as well as their experiences of distress. The first set of interviews (*n* = 21), which took place before the survey, were utilised as an initial exploration of the topic. The interview data informed the range of demographic data collected; the remaining survey items employed validated instruments which remained unchanged. Survey participants were asked to provide an email address if they were interested in participating in a follow-up interview. Those junior doctors whose survey results indicated severe depression and/or anxiety on DASS-21 [32] or high suicidality of Paykel’s measure [33] and who had consented were contacted via email to ask if they would like to take part in the second set of interviews (*n* = 15), which explored concerns that emerged from the survey in more depth. We sampled doctors with higher levels of distress via the survey as this group was under-represented in the first wave of interviews. The results of our mixed methods findings have been published [34]; the interview data elucidate participants’ responses to survey items related to specific working conditions and cultures which contribute to stress/distress. This paper reports on findings from the qualitative data only.

Interested individuals contacted FK or JS and gave informed consent. Thirty-six junior doctors working in the NHS in England and Wales (31 = female) were interviewed.

Stakeholders were invited to the meeting via email. We publicised the workshop via specialist schools and social media. Invited stakeholders were a variety of healthcare professionals (including junior doctors), undergraduate medical school leads, postgraduate speciality school leads and NHS policymakers. Interview participants, representatives from key interest groups including the Society of Occupational Medicine, the Academy of Medical Royal Colleges, Royal College of Physicians, specialist mental health service providers, policymakers from Health Education England and other key NHS organisations with a responsibility specifically for doctors’ working conditions and wellbeing were all invited.

All participants and stakeholders gave informed consent prior to taking part in the interview or survey. Ethical approval was granted by the relevant university board and the Health Research Authority (reference number: 19/HRA/6579).

### Analysis

Data were analysed by JS and FK using thematic analysis [35, 36]. Analysis began once all interviews had been conducted. FK analysed the first set of qualitative interviews (*N* = 21), while JS analysed the second set (*N* = 15). Transcripts were analysed one by one using NVivo 12. As the inductive analysis progressed, a table of emergent themes was developed and refined. Each new transcript led to new codes and themes being added or expanded. In addition, RR read and coded a sub-sample (*n* = 11) of the initial 21 transcripts, while a further three members of the team (MB, AT, CCG) read and fed back on six interviews. Their views and insights were incorporated into the NVivo codes. JS refined these codes to create relevant tables of themes once all interviews had been analysed and discussed. We do not consider data saturation, a concept which arises from grounded theory and which we did not employ in the study, to be relevant to our thematic analysis [37].

Relevant notes from the stakeholder meeting were then read carefully and coded according to the table of themes arising from analysis of the interview data. This table of themes was used as a coding schema to explore the data from the stakeholder meeting, meaning this second stage of the analysis was more deductive than inductive. It was found that stakeholder recommendations raised (and built on) similar issues to the qualitative findings, as described in more detail in the findings section.

### Findings

All interviewed participants had suggestions for how their working conditions and cultures could be improved to alleviate distress. Recommendations broke down into two broad themes: organisational recommendations and interpersonal recommendations, which are presented here. We also recap the answers stakeholders gave to our three questions.

### Organisational recommendations

Many recommendations were made that could be applied at an organisational level and thus acted upon by trusts, employers or those who train doctors. These suggestions were focused on resources and rotas.

#### Resources

A major organisational recommendation pertained to the importance of providing basic environmental resources for use while doctors were at work. Research participants and stakeholders agreed that the practical resources in place for junior doctors are often inadequate. One stakeholder, a foundation school director, reported having been asked to sleep on an old airbed during a night shift, which led to a discussion between several doctor stakeholders about the importance of getting the basics right – including providing enough food, forks and toilet paper (all of which were often lacking) as well as a space to sleep. Participants also reported that, even before the pandemic, doctors’ messes had been removed, making it hard to relax with colleagues:

*In the new contract it states if you're too tired to drive home they should give you accommodation [ … ] or arrange transport home for you, but I’ve only seen that in one hospital. (JD34)*

*… there's nowhere for doctors you know sort of pre-COVID to sort of socialise and get to know each other and break down those you know barriers of the hierarchy. (JD27)*

The stakeholders posited that conditions like this imply that staff are expendable. Therefore, organisations are advised to keep or reinstate safe spaces to relax. Indeed, when such resources were available, study participants appreciated them:

*So, the one hospital I worked at earlier this year that had a trainee room, we all had a key to it [ … ] that was brilliant, cos we would all go and have our lunch in there. (JD29)*

Correct resource levels were also important in terms of staffing, although difficulties around organisations achieving this were acknowledged:

*… it would just fix working conditions [ … ] making sure it's adequately staffed [ … ] it's such an easy thing to say, but I know that it's very difficult to, to actually organise. (JD24)*

Stakeholders agreed with this recommendation. One NHS director stated that there are not enough staff on acute wards, meaning that junior doctors feel unsupported, while a consultant stated when you solve staffing issues, much distress abates.

Improvements to training resources for staff, starting from medical school, were frequently recommended by the research participants. Participants emphasised that organisations should provide more education about mental health and burnout. One junior doctor participant recommended that organisations should teach doctors how to grieve and find support when patients die, while another felt that doctors living with disabilities would benefit from more specific training about how to negotiate their roles and related logistics.

*… disabled people spend most of their time finding ways around the system and figuring out ways to do stuff. [ … ] advice on how to do that and ways to support themselves through that process (JD31)*

Many participants made the point that it was hard to recognise symptoms in themselves without this training:

*… there needs to be teaching and training on “what does struggling look like”, cos I think a lot of people are just like, “I'm just tired”. It's like, you know, there's a difference between “I'm just tired” versus “I haven't slept in a week, and I'm not coping”. (JD26)*

One stakeholder, a senior Health Education England (HEE) employee, stated that a “massive reform” of education and training is needed. Two others (a DME at an English hospital and a foundation school head) suggested that longer periods following medical school, including internships, might decrease junior doctors’ anxiety.

#### Rotas

Participants suggested changes to rotas and timetables to reduce stress. Several mentioned how useful it would be for organisations to provide protected admin time for junior doctors, as they do for consultants, while participants also felt that having their rota placements, hours and annual leave confirmed further in advance would be useful. Another suggested that an electronic rota system might make things easier:

*It's just fully expected that you'll work your 10-hour day and then you'll go home and you'll do another hour or two of revision or portfolio stuff or anything like that. And I don't know where they expect us to get the time from and not burn out. (JD28)*

*… it would be great if they could give me the rota a year in advance so I could plan my life by weekends. Instead of giving it us six weeks before the four-month rotation starts. (JD34)*

*I struggle with keeping track of how many people I’ve emailed to arrange a rota swap and things like that [yeah], whereas an electronic system can kind of be streamlined and make it a bit easier. (JD07)*

Stakeholders agreed that organisations could make beneficial changes to rotations and rotas. Some suggested increasing the length of rotations, whilst others (a senior HEE employee and an A&E consultant) were already trying to keep rotations local and more family-friendly.

### Interpersonal recommendations

As well as these organisational recommendations, many junior doctors made interpersonal suggestions for improving their working conditions. These recommendations were focused on communication and support/teamwork.

#### Communication

Research participants felt that clearer interpersonal communications would foster teamwork and so lead to better working conditions. One reported having been left dangerously unsupported after her supervisor went off sick while she was in her first year. She felt that better communication would prevent such situations in future:

*I think a general kind of guideline would be good what to expect here in the NHS as an employee. That I knew that I should have a supervisor, who is present, I shouldn’t be on my own. (JD36)*

The introduction of a whistleblowing system was also suggested as beneficial, so that doctors could safely communicate any concerns before actions were required:

*… everywhere needs to, erm have a kind of, robust whistleblowing process, erm, where you can raise concerns without, cos I think (sigh), the fear of repercussion is a massive thing for people. (JD22)*

One of the stakeholders, a professor of psychiatry, highlighted the ‘Freedom to Speak Up’ Guardians, who operate in every NHS trust, theoretically enabling such beneficial communication. This service is confidential and aims to help those who want to point out flaws in the system. However, as evidenced above, it was acknowledged that many doctors do not know that this service exists or would not feel confident using it.

Receiving positive feedback for their efforts was cited as an interpersonal change that could improve junior doctor participants’ working conditions and cultures, and hence their confidence, self-worth and wellbeing.

*Just feeling like you're not just a number or you know a mailing list on the rota team's um computer system that someone knows you and appreciates that oh you've stayed two hours late three days in a row, like, wow, thank you. (JD27)*

Several stakeholders stated that Datix, a web-based incident reporting system, can be used as a threatening way to control the workforce, although others felt that when Datixes are done well, they can be a learning tool. One deputy DME at an English hospital introduced the idea of ‘Greatix’, a system for documenting positive comments about staff, which are then reviewed alongside Datix comments in risk meetings.

Many of the research participants described situations in which mental ill-health was stigmatised to the degree where it could not be discussed. Instead, they proposed a culture in which it was acceptable and encouraged for healthcare professionals, especially senior members of staff, to communicate about mental health struggles and challenges.

*… More senior doctors need to be open about their struggles and their issues in training because I think there’s a perception that you need to be invulnerable among more junior trainees in particular, which I think as people gradually have breakdowns, they’re robbed of that illusion. (JD07)*

*I wonder whether if people talked about stuff more freely and it became part of the culture in medical school, whether that means that we would then have a whole generation of doctors who are not too scared to talk about it. (JD11)*

Several participants gave examples of times when being able to speak freely about mental health difficulties had led to improvements:

*… When you actually sit down and have a good conversation with people, you realise everybody’s in the same boat. (JD22)*

*… If anybody even mentions you know that they're struggling a bit I go well you know what?! To try and like normalise it a bit you know just to say no it's fine, you know you go through this stuff and you know and I’m functioning and I’m okay now [ … ] But I wouldn’t be if I hadn’t got help. (JD32)*

In line with this, one stakeholder (who was also a junior doctor) discussed the importance of employers honestly managing junior doctors’ expectations in terms of the impact of the work and offering support when it is needed. Another junior doctor stakeholder felt that there are ‘pockets’ of gold standard practice around communication within the NHS. These should be highlighted and showcased so that other teams can learn from them.

#### Support and teamwork

As reported in another of our papers, several participants in this study were appreciative of the more consistent teams they worked in during COVID-19 [26]. One stakeholder, a senior HEE employee, made the point that this response to working during the pandemic demonstrated the possibility of working more inclusively at all times. Unfortunately, such positive changes often ended after the first wave of the pandemic. The stakeholders recommended that leaders might wish to focus less focus on portfolio building for junior doctors and more on fostering teamwork.

Many junior doctor participants felt that both supervisory and peer group support could be used to improve their interpersonal working experience. One felt that a supportive element was missing from supervision:

*I would really like to see sort of the assessing and policing elements of supervision split from the supportive elements of it. I think doctors have all sorts of assessments but what they don’t get so much of is that supportive element built into their training and that’s the really important stuff. (JD30)*

Similarly, several stakeholders suggested that the clinical debrief sessions which take place in some medical schools could also be utilised for junior doctors. One senior HEE employee suggested mental health check-ins at the start of each job. Stakeholders felt that compassionate leadership and care for the workforce, through supervision, were just as important as providing more doctors. One medical professor felt that the high prevalence of bullying reported in the NHS [38, 39] was an indication of how unhappy the workforce is.

Study participants felt that as well as support itself needing to improve, it needed to be more accessible to junior doctors. Several made practical suggestions about this, such as putting structured processes and plans in place to monitor and support their mental health. When positive supervision and peer-to-peer support were in place, participants experienced them as helpful:

*… people don't want to go hunting for help. They need help to be shoved in their face. Uh, and that will make conditions better. (JD26)*

*For example, if, as junior doctors, you saw a therapist once a month… if we made it universal (which is very easy to say) that for one hour a month, everyone had to see someone to talk to, I think that would be a good way of moving forward. (JD03)*

*… the thing which actually pushed me to take time off when I was unwell was my supervisor checking in, erm, making sure that not just clinically and professionally, but personally, that you’re doing OK. (JD22)*

### Responses to the stakeholder questions

As stated in the Methods section, stakeholders were divided into smaller groups to consider three specific questions. We will recap those questions and the answers that were fed back to the main group here.

#### What power do individuals or organisations have to make changes?

Some stakeholders felt it was crucial to try to retain senior members of staff who could provide a ‘parental role’ for the team. However, others felt that while junior doctors may believe consultants have the power to affect change, consultants themselves feel their power is limited, despite their greater influence. One medical director felt that it is virtually impossible to change culture from the ground up and that the only way to make change is via legal means – that is, by putting sanctions on trusts. Several stakeholders made the point that everyone in a team needs to be on board to affect change, suggesting that perhaps the power lies in teams and organisations rather than individuals.

#### How can individuals or organisations make realistic improvements to prevent work-related distress and support junior doctors?

The director of a mental health organisation stated that she would feed back the results of the stakeholder meeting to both medical schools and educators to try to ensure that awareness is being raised at all levels. As mentioned above, one junior doctor emphasised the importance of managing new doctors’ expectations to try to prevent distress by being realistic about what the role entails. Similarly, others felt that providing more transparency during training might help alleviate distress.

#### What actions can individuals or organisations take to address the causes of work-related distress identified by this research?

Stakeholders discussed the importance of getting basic resources right. As outlined above, many basic needs such as food, a place to rest or sleep, toilet roll or cutlery are not currently provided for doctors; addressing this was seen as crucial. The importance of recruiting and retaining staff and increasing staff ratios were discussed, as well as the need for clear pathways for support when required. The next step for us or other researchers might be to determine which organisations could address which of these recommendations.

## Discussion

We asked junior doctors for recommendations for improving their working conditions and cultures. We interviewed 36 junior doctors and held a stakeholder meeting with healthcare professionals (including junior doctors), undergraduate medical school leads, postgraduate speciality school leads and NHS policymakers. As such, this paper makes a further contribution to the previous work arising from this overall study [17, 26, 27, 34] by including tangible recommendations for improving junior doctors’ working conditions.

Findings split into two themes: organisational and interpersonal recommendations. These recommendations come from both the research participants (those on the frontline) and the stakeholders (those who may have the power to effect change), leading to a co-productive, reciprocal outcome [29, 30] which we hope will impact policy. As noted in the method section, some of the interviews were conducted during the COVID-19 pandemic, which bought about many changes to the working conditions of junior doctors [26]. We believe that the ability to compare conditions before and during the pandemic has strengthened our findings by demonstrating both positive (consistent teams) and negative (less space to socialise and relax) changes which occurred during this time.

Research participants and stakeholders gave recommendations for how organisations could improve junior doctors’ working conditions. These included improvements to training and teamwork as well as changes to hours and increased resources. It is known that healthcare teams in the UK are understaffed [1, 3]. While short staffing and rota gaps are challenging issues which may need addressing at a governmental level, participants and stakeholders had further recommendations which could be applied by managers, employers and policymakers. Clarity and responsiveness to organisational processes that are easier to address (such as responding to leave requests, policies on supervision, guidelines around what support to expect and advertising the support that is on offer) can help junior doctors to feel more in control of their working environment. This should be an area to focus on for teams across the NHS, as clarity and responsiveness in processes may substantially improve junior doctor wellbeing.

Doctors felt that training in recognising and managing mental health symptoms in themselves and their colleagues would be beneficial. Currently, efforts are being made to tackle this subject in medical school, although limitations to engagement have been reported [40]. We recommend that training in this area is reviewed during both medical school and throughout postgraduate training, with an emphasis placed on the importance of recognising symptoms, seeking help and undertaking self-care in a way which de-stigmatises this issue. While this is not a complete solution to the many challenges of working within the NHS, it may go some way to providing extra support for distressed doctors. Such training would need to be carefully designed in conjunction with doctors-in-training to ensure it meets the needs of the population it is intended to serve.

Participants and stakeholders reported a lack of transparency around mental ill-health in the NHS. This is likely to be connected to the stigma which is still attached to mental health concerns, both in UK society in general [41, 42] and within NHS staff in particular [43], where a ‘culture of invulnerability’ [44, 45] demands health and perfection at all times. The more that doctors feel supported in being able to speak out about their distress and encouraged by senior members of staff (such as supervisors), the more that stigma should ease, suggesting again the importance of compassionate leadership and supervision.

Given the commonly reported toxic working culture of the NHS [17, 27], where lack of support and bullying [38, 39, 46] are frequent, it is unsurprising that both the participants and stakeholders in the current study felt that support could be improved. Previous researchers have demonstrated the benefits of formalised support such as Balint groups [47] and informal, peer support for junior doctors in particular [48, 49]. Such support could be of widespread benefit for doctors working across the NHS.

The benefits of more consistent working teams for healthcare professionals have been reported by other authors [50], while inconsistent teams make it harder for doctors to feel supported [44]. As one stakeholder in our study noted, COVID-19 has demonstrated that positive changes can be made in terms of organising effective teams, so we call on employers and policymakers to work towards this in the future.

As mentioned earlier, this paper is an important contribution to the literature as it provides tangible, realistic recommendations for improving working conditions for junior doctors. However, in addition to this, the wide range of topics covered by both research participants and stakeholders highlights the need for participatory, collaborative approaches to solutions, which we argue have been modelled by our stakeholder meeting and this article. We suggest that employers, organisations and teams need to work together to implement the recommendations, which are summarised below.

### Recommendations

Based on our findings, we make the following organisational recommendations, which could be implemented by team managers:

- Consideration should be given to how consistent teams could be maintained during rotations and on-call shifts as far as possible
- Hospitals that practice high standards of communication be showcased so that others can learn from them
- Junior doctors given, where possible, protected self-development time as part of their rota, as has been successfully introduced in specialities such as paediatrics
- Formal and informal support/clinical supervision (such as peer or Balint groups) put in place and made accessible
- Open discussion of mental health challenges and experiences encouraged at all levels and demonstrated by senior leaders
- Junior doctors given positive, as well as constructive, feedback whenever appropriate

We also make the following recommendations to employers and those who train doctors:

- Where possible, every effort should be made to adhere to standards around levels of staffing that are set out in junior doctors’ contracts
- Training around how to recognise and seek help for mental health problems be improved in medical school and beyond, including signposting at induction for how to seek help in each new rotation
- Ensuring doctors’ messes and improved resources for resting (including space for napping during overnight shifts), eating and drinking are provided

As stated in the introduction, we have known for a long time that doctors, including junior doctors, are more vulnerable to mental ill-health because of the pressures placed on them. As such, it is time for action. Whilst our recommendations may, at times, feel like ‘common sense’, they are areas and suggestions which are lacking for many junior doctors. Employers, managers and policymakers have a duty of care to look after their staff; we call on those people to listen to what the participants in this study have to say.

### Strengths and limitations

We teamed our in-depth, qualitative interviews with 36 junior doctors with findings from a stakeholder meeting at which healthcare professionals (including junior doctors), undergraduate medical school leads, postgraduate speciality school leads and NHS policymakers were present. As such, the recommendations in this paper are grounded in the stories and experiences of those at the frontline of the NHS at various levels. We suggest that this co-productive [29, 30, 51] approach offers other researchers a template for getting findings into ‘the real world’ and in front of the eyes of people who have the power to make a difference.

While this paper has important strengths, it also has a limitation in that the participant group was heavily weighted towards female doctors, so might not be as applicable to male doctors. However, there are particular concerns over female doctors based on sex differences in published suicide rates [11]. It may be that there are also differences between male and female doctors in key drivers of risk. Therefore, we suggest that authors of future studies should aim to clarify any such differences.

Further to this, as with all research, the experiences and beliefs of the researchers may have influenced their interpretation of the results. Finally, more research is needed into who could act upon the recommendations made in this paper and whether they do improve working conditions and culture for junior doctors.

## Supplementary Information

**Additional file 1: **COREQ checklist for ‘Recommendations for improving the working conditions and cultures of distressed junior doctors, based on a qualitative study and stakeholder perspectives’.

## Data Availability

The datasets generated and/or analysed during the current study are not publicly available as we do not have permission from ethics nor participants to share this sensitive data, but are available from the corresponding author on reasonable request.
