# Supplementary material for: Recommendations for improving the working conditions and cultures of distressed junior doctors, based on a qualitative study and stakeholder perspectives
Source: BMC Health Serv Res. 2022 Nov 10;22:1333. doi: 10.1186/s12913-022-08728-2 (PMC9647238; doi:10.1186/s12913-022-08728-2)
Supplement: Supplementary file 1 — Additional file 1: COREQ checklist for ‘Recommendations for improving the working conditions and cultures of distressed junior doctors, based on a qualitative study and stakeholder perspectives’. [file 12913_2022_8728_MOESM1_ESM.docx]

COREQ checklist for ‘Recommendations for improving the working conditions and cultures of distressed junior doctors, based on a qualitative study and stakeholder perspectives’

Developed from: Tong, A., Sainsbury, P., & Craig, J. [2007]. Consolidated criteria for reporting qualitative research [COREQ]: a 32-item checklist for interviews and focus groups. *International journal for quality in health care*, *19*[6], 349-357.

| **No. Item** | **Guide questions/description** | **Reported on page #** |
| --- | --- | --- |
| **Domain 1: Research team and reflexivity** |  |  |
| *Personal characteristics* |  |  |
| 1. Interviewer/ facilitator | Which author/s conducted the interview or focus group? | Page 8 |
| 2. Credentials | What were the researcher’s credentials? E.g. PhD, MD. | Page 8 |
| 3. Occupation | What was their occupation at the time of the study? | Page 8 |
| 4. Gender | Was the researcher male or female? | Page 8 |
| 5. Experience and training | What experience or training did the researcher have? | Page 8 |
| *Relationship with participants* |  |  |
| 6. Relationship established | Was a relationship established prior to study commencement? | Page 8 |
| 7. Participant knowledge of interviewer | What did the participant know about the researcher? E.g. personal goals, reasons for doing the research. | Page 8 |
| 8. Interviewer characteristics | What characteristics were reported about the interviewer/facilitator? E.g. bias, assumptions, reasons and interests in the research topic | Page 10 |
| **Domain 2: study design** |  |  |
| *Theoretical framework* |  |  |
| 9. Methodological orientation and theory | What methodological orientation was stated to underpin the study? E.g. grounded theory, discourse analysis, ethnography, phenomenology, content analysis | Page 11 |
| *Participant sampling* |  |  |
| 10. Sampling | How were participants selected? E.g. purposive, convenience, consecutive, snowball | Pages 9 & 10 |
| 11. Method of approach | How were participants approached? E.g. face-to-face, telephone, mail, email | Pages 9 & 10 |
| 12. Sample size | How many participants were in the study? | Page 9 |
| 13. Non-participation | How many people refused to participate or dropped out? Reasons? | None |
| *Setting* |  |  |
| 14. Setting of data collection | Where was the data collected? E.g. home, clinic, workplace | Page 7 |
| 15. Presence of non-participants | Was anyone else present besides the participants and the researchers? | No |
| 16. Description of the sample | What were the important characteristics of the sample? E.g. demographic data, date | Pages 9 & 10 |
| *Data collection* |  |  |
| 17. Interview guide | Were questions, prompts, guides provided by the authors? Was it pilot tested? | Pages 7 & 8 |
| 18. Repeat interviews | Were repeat interviews carried out? If yes, how many? | No |
| 19. Audio/visual recording | Did the research use audio or visual recording to collect the data? | Page 8 |
| 20. Field notes | Were field notes made during and/or after the interview or focus group? | Page 8 |
| 21. Duration | What was the duration of the interviews or focus group? | Page 8 |
| 22. Data saturation | Was data saturation discussed? | Page 11 |
| 23. Transcripts returned | Were transcripts return to participants for comment and/or correction? | No, due to lack of resources |
| **Domain 3: analysis and findings** |  |  |
| *Data analysis* |  |  |
| 24. Number of data coders | How many data coders coded the data? | Page 11 |
| 25. Description of the coding tree | Did authors provide a description of the coding tree? | No |
| 26. Derivation of themes | Derived from the data? | Pages 11-22 |
| 27. Software | What software, if applicable, was used to manage the data? | Page 11 |
| 28. Participant checking | Did participants provide feedback on the findings? | No, due to lack of resources |
| *Reporting* |  |  |
| 29. Quotations presented | Were participant quotations presented to illustrate the themes/findings? Was each quotation identified? E.g. participant number | Pages 11-22 |
| 30. Data and findings consistent | Was there consistency between the data presented and the findings | Yes, see pages 11-22 |
| 31. Clarity of major themes | Were major themes presented in the findings? | Yes |
| 32. Clarity of minor themes | Is there a description of diverse cases or discussion of minor themes? | Yes |
